# Renal expression of Bach1 and oxidative stress in chronic kidney disease: evidence from a preclinical model

**DOI:** 10.1590/2175-8239-JBN-2025-0333en

**Published:** 2026-06-12

**Authors:** Beatriz Oliveira Da Cruz, Patricia Pereira Almeida, Nathalia Silva-Costa, Michele Lima Brito, D’Angelo Carlo Magliano, Milena Barcza Stockler-Pinto

**Affiliations:** 1 Universidade Federal Fluminense, Programa de Pós-Graduação em Ciências Cardiovasculares, Niterói, RJ, Brazil.; 2 Universidade Federal Fluminense, Programa de Pós-Graduação em Patologia, Niterói, RJ, Brazil.

**Keywords:** Kidney disease, Oxidative Stress, Antioxidant Response Elements, Nephrectomy.

## Abstract

**Introduction:**

The transcription factor BTB and CNC homology 1 (Bach1) represses the nuclear factor erythroid 2–related factor 2 (Nrf2), which controls antioxidant gene expression, and its role in chronic kidney disease (CKD) remains unclear.

**Methods:**

CKD was induced by 5/6 nephrectomy in rats. Kidney fibrosis and oxidative stress markers were measured. The gene expression of Nrf2, Bach1, and nuclear factor kappa B (NF-κB) was assessed.

**Results:**

CKD increased oxidative stress markers in plasma, kidney, and heart, as well as promoted kidney fibrosis. Moreover, CKD reduced cardiac Nrf2 expression. However, Bach1, Nrf2, and NF-κB remained unchanged in the kidney.

**Conclusion:**

CKD did not modulate Bach1 mRNA levels in the kidneys of 5/6 nephrectomized rats.

## INTRODUCTION

Reactive oxygen species (ROS) generation in chronic kidney disease (CKD) con­tributes to disease pathophysiology through direct renal cell injury, leading to functional impairment, inflammation, and fibrosis, as well as through the activation of signaling pathways and transcription factors that amplify these processes^1^. BTB and CNC homology 1 (Bach1) is a widely expressed trans­cription factor and a key regulator of the oxidative stress response^2^. Bach1 inhibits the nuclear factor erythroid 2–related factor 2 (Nrf2) by competing for the antioxidant response element, thereby suppressing antioxidant gene expression^2^,^3^ and contributing to increased ROS, apoptosis, and cellular senescence^4^. While Nrf2 mediates cytoprotective responses, Bach1 acts as its primary negative regulator. Furthermore, the balance between oxidative stress and inflammation is regulated by crosstalk between the nuclear factor kappa B (NF-κB) and Nrf2 pathways, as Nrf2 activation can attenuate NF-κB–mediated inflammation, whereas NF-κB activation may suppress the antioxidant response^5^. Aging-associated increases in Bach1 impair redox homeostasis and exacerbate oxidative stress. In CKD, reduced Nrf2 expression and chronic inflammation are frequently observed. Thus, Bach1 inhibition may enhance Nrf2 activity and represent a potential therapeutic strategy^2^,^3^,^4^,6>.

Accordingly, this study evaluated oxidative stress, inflammatory markers, and renal structural alterations in a preclinical model of CKD.

## METHODS

All procedures were conducted in accordance with the ARRIVE guidelines and complied with the criteria established by the National Council for the Control of Animal Experimentation (CONCEA) and were approved by the Animal Research Ethics Committee of the *Universidade Federal Fluminense* (protocol number 956/2017).

### Animals

Fourteen male Wistar rats were used and housed under controlled temperature (22 ± 1 °C) and humidity (60 ± 10%) conditions, with a 12:12 h light–dark cycle. Animals had free access to commercial chow and water and were weighed weekly. CKD was induced by 5/6 nephrectomy, as previously described^7^. The animals were randomly assigned to two groups: a control (Sham) group, which underwent surgical manipulation of the renal pedicles without nephrectomy (n = 7), and a CKD group subjected to 5/6 nephrectomy (n = 7).

### Euthanasia

Animals were deeply anesthetized with ketamine (40 mg/kg) and xylazine (8 mg/kg) (*Laboratório Virbac S.A.*, São Paulo, Brazil) and euthanized by cardiac puncture until total exsanguination. Kidney and heart tissues were carefully dissected and stored at -80 °C for further analysis.

### Biochemical Analysis

Blood samples were drawn from each animal by cardiac puncture. Plasma and serum were separated by centrifugation (15 min, 4500 × g, 4 °C). Serum levels of creatinine and urea were measured using Bioclin^®^ kits with the Bioclin BS-120 chemistry analyzer.

### Lipid Peroxidation and Protein Carbonylation

Lipid peroxidation in renal and cardiac tissues was evaluated by the thiobarbituric acid reactive substances (TBARS) assay, and plasma protein oxidation was assessed by quantifying carbonyl groups using 2,4-dinitrophenylhydrazine (DNPH). Both analyses followed the methodology described by Costa et al.^8^. All samples were prepared in RIPA buffer containing a protease inhibitor cocktail (Sigma-Aldrich, Missouri, USA).

### Kidney Morphology

Kidney sections were stained with Masson trichrome to evaluate tubulointerstitial fibrosis. Ten random fields of the tubulointerstitial area from five animals were selected and analyzed using the Image-Pro Plus (Media Cybernetics, Silver Spring, MD, USA) through the density threshold selection tool.

### Gene Expression

*Bach1*, *NF-*κ*B,* and *Nrf2* mRNA expression was evaluated using real-time quantitative polymerase chain reaction (PCR), according to Leal et al.^9^. TaqMan Gene Expression Assays (Thermo Fisher Scientific) were used for *Bach1* (Rn01477344_m1), *NF-*κ*B* (Rn01399572_m1), *Nrf2* (Rn00477784_m1), and the control gene, *Gapdh* (Rn01775763_g1). Reactions were performed on the ABI Prism 7500 Sequence Detection System (Applied Biosystems) under standard conditions. *Bach1* expression was evaluated only in kidney tissue, and *NF-*κ*B* and *Nrf2* were evaluated in the kidney and heart.

### Statistical Analysis

Results are presented as mean ± standard deviation, and the ROUT test was used to identify outliers. Data normality was assessed with the Shapiro–Wilk test. Group comparisons used an unpaired *t-*test, and a two-way ANOVA was applied for analyses over time. A multivariate approach was also employed. The variation in sample size for gene expression reflected limited biological material. A correlation matrix was generated, and principal component analysis (PCA) was conducted on standardized variables (z-score transformation) to account for differences in measurement scales and ensure equal contribution of all variables. Component retention followed the Kaiser–Guttman criterion, with eigenvalues ≥ 1.0 considered significant, and the variance explained by each retained component was reported. PCA constituted an exploratory, unsupervised dimensionality reduction method to identify dominant patterns and relationships among correlated variables, facilitating data interpretation and visualization rather than inferential testing. All analyses were performed using GraphPad Prism (version 10.2.3; GraphPad Software, Boston, MA, USA), and statistical significance was set at *p* < 0.05.

## RESULTS

**Figure 1:**
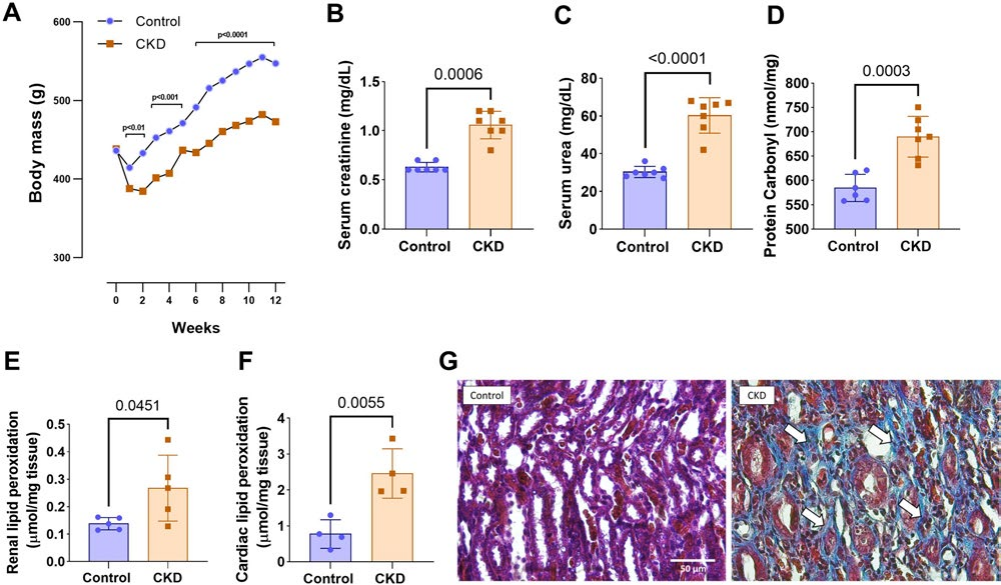
Body mass evolution over 12 weeks in control and CKD groups (A). Serum creatinine (B), serum urea (C), and protein carbonyl (D). Kidney (E) and heart (F) Lipid peroxidation levels in control and CKD animals. Representative photomicrographs of renal tissue from control and CKD animals (G). Histological analysis using Masson’s trichrome staining (×400 magnification) to evaluate tubulointerstitial fibrosis. Arrows (↑) indicate collagen deposition, markedly increased in the CKD group compared to the control group. (A), (F), and (G): control: n = 5; CKD: n = 5; (B) and (C): control: n = 7; CKD: n = 7; (D) control: n = 6; CKD: n = 7; (E): control: n = 4; CKD: n = 4.

After 4 weeks, CKD was established. The CKD group showed significantly lower body mass than the control group (Figure 1A). As expected, the CKD group presented a significant increase in creatinine (1.06 ± 0.14 vs. 0.63 ± 0.05 mg/dL; p = 0.0006) and serum urea levels compared to the control group (60.29 ± 5.70 vs. 30.29 ± 2.98 mg/dL; p < 0.0001) (Figures 1B and 1C). Additionally, plasma protein carbonyl levels were significantly elevated in the CKD group when compared to the control group (689.90 ± 42.06 vs. 584.80 ± 27.73 nmol/mg protein; p = 0.0003) (Figure 1D). Regarding lipid peroxidation, the CKD rats exhibited markedly higher levels in the kidney (0.27 ± 0.12 vs. 0.14 ± 0.02 μmol/mg tissue; p = 0.0451) and heart (2.46 ± 0.69 vs. 0.77 ± 0.40 μmol/mg tissue; p = 0.0055) compared to the control group (Figures 1E and 1F). Histological analysis of kidney photomicrographs revealed a marked increase in tubulointerstitial fibrosis in the CKD group relative to the control group (Figure 1G).

Abbreviations – CKD: chronic kidney disease.

Notes – Data are presented as mean ± SD. Two-way ANOVA followed by the Bonferroni post hoc test was used for body mass evolution; the unpaired t-test (parametric data) was applied for the remaining parameters.

**Figure 2:**
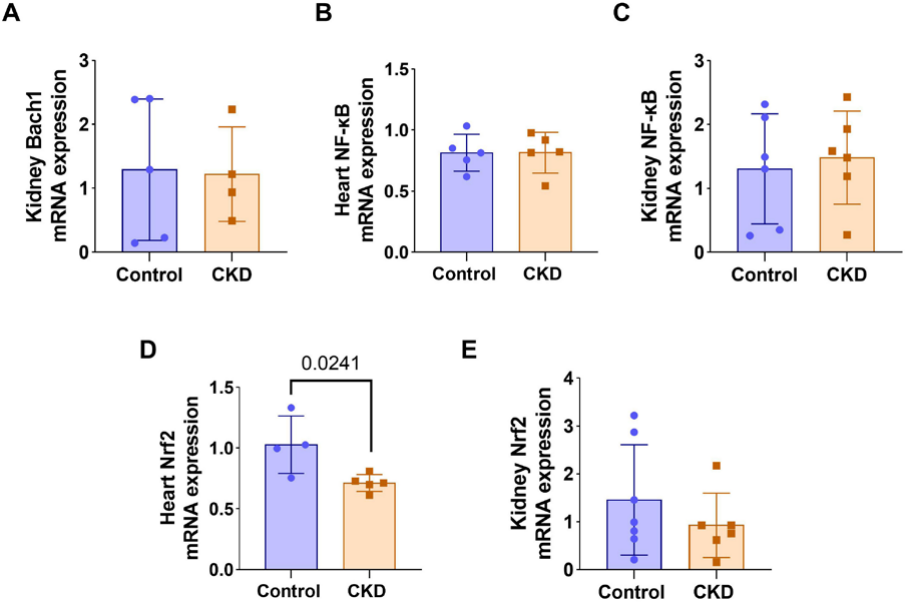
mRNA expression of *Bach1* in the kidney (A), *Nf*-κ*B* in the heart (B), *Nf*-κ*B* in the kidney (C), *Nrf2* in the heart (D), and *Nrf2* in the kidney (E) in the experimental groups.

There was no significant difference in renal *Bach1* mRNA expression between groups (Figure 2A). *NF-*κ*B* mRNA expression remained unchanged in both heart (p = 0.9982) and kidney (p = 0.7117) tissues (Figures 2B and 2C). However, animals in the CKD group showed lower cardiac *Nrf2* mRNA expression (0.71 ± 0.07 vs. 1.03 ± 0.24; p = 0.0241) compared to the control group (Figure 2D), whereas no difference was observed in the kidney (1.46 ± 1.53 vs. 0.92 ± 0.67; p = 0.3432) (Figure 2E).

Abbreviations – *Bach1*: BTB and CNC homology 1; *Nf*-κ*B*: nuclear factor kappa B; and *Nrf2*: nuclear factor erythroid 2-related factor 2. CKD: chronic kidney disease.

Notes – Statistical significance: p < 0.05. Unpaired Student’s t-test; outliers identified using the ROUT method were excluded from the statistical analysis. (A): control: n = 5; CKD: n = 4; (B): control: n = 7; CKD: n = 6; (C): control: n = 6; CKD: n = 6; (D): control: n = 4; CKD: n = 5; (E): control: n = 5; CKD: n = 5.

**Figure 3:**
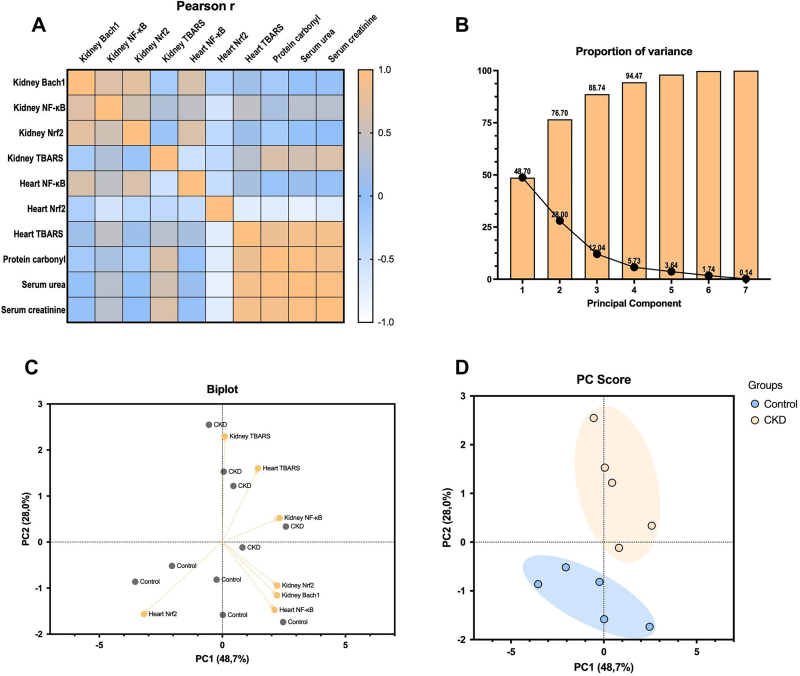
Principal component analysis. Pearson’s correlation heatmap (A). Cumulative variance of PCs (B). Biplot of loading scores (C). Score plots of PCs (D). Control: n = 5; CKD: n = 5.

Multivariate analysis considered parameters related to inflammation, oxidative stress, and renal function. The heatmap matrix is presented in Figure 3A. PCA yielded two principal components, explaining 48.7% (PC1) and 28.0% (PC2) of the variance, accounting for a cumulative variance of 76.7% (Figure 3B). PC1 was strongly correlated with renal *Bach1*, *NF-*κ*B*, and *Nrf2* expression, indicating an integrated response among these variables. PC2 was predominantly associated with renal lipid peroxidation levels and cardiac *Bach1*, *NF-*κ*B*, and *Nrf2* expression (Figures 3C and 3D).

Abbreviations – CKD: chronic kidney disease; PC: principal component.

## DISCUSSION

The results of this study demonstrate that CKD in rats is associated with increased oxidative stress, as evidenced by elevated lipid peroxidation in renal and cardiac tissues and increased plasma protein carbonyl levels. In addition, CKD animals exhibited reduced Nrf2 mRNA expression in cardiac tissue, suggesting an impaired systemic antioxidant response. Notably, *Bach1* mRNA expression in the kidney did not differ significantly between groups, despite its established role as a negative regulator of Nrf2.

As activation of the Nrf2 pathway represents a therapeutic target in CKD, its downregulation has been associated with the progression of cardiometabolic disorders^10^,^11^. Nrf2 regulation involves complex molecular mechanisms influenced by multiple pathophysiological processes^12^. In the present study, Nrf2 mRNA levels differed between tissues, with reduced expression in the heart but not in the kidney, indicating tissue-specific regulation that may be associated with increased lipid peroxidation and protein carbonylation.

Despite the presence of CKD and associated oxidative stress and inflammation in our experimental model, no significant differences in renal Bach1 gene expression were observed between groups. This finding was unexpected, given that Bach1 is a redox-sensitive transcriptional repressor known to respond to oxidative stress by regulating antioxidant response elements, often acting in opposition to Nrf2^4^,^13^. Previous studies have shown that, under acute oxidative conditions, such as rhabdomyolysis-induced acute kidney injury, Bach1 expression and subcellular localization can change dynamically, with nuclear export representing an early response to elevated heme levels^14^. In contrast, under chronic conditions such as CKD, Bach1 regulation may differ and be influenced by adaptive or compensatory mechanisms that help maintain stable expression levels despite sustained oxidative stress^3^,^15^. Thus, the absence of transcriptional changes in Bach1 does not negate its potential functional relevance, as its activity may be modulated by post-transcriptional and post-translational mechanisms, including changes in protein stability or subcellular localization^16^. Although the Bach1–Nrf2 interaction may be dynamically regulated over time, this study was limited to a single experimental time point; therefore, any temporal interpretation should therefore be made with caution. In addition, in the 5/6 nephrectomy model, increased tubulointerstitial fibrosis is observed, along with adaptive hyperfiltration and glomerular hypertrophy of residual nephrons to sustain excretory capacity^17^. These compensatory changes increase intraglomerular pressure and mechanical stress, promoting filtration barrier injury, oxidative stress, and activation of pro-inflammatory pathways^18^. Importantly, inflammatory activity may occur without changes in NF-κB gene expression, as NF-κB activation is largely regulated at the post-translational level^19^. Collectively, these mechanisms may have influenced renal Bach1 expression and impaired antioxidant responses, including Nrf2.

Finally, PCA analysis demonstrated that PC1 explained 48.7% of the total variance and represented the main axis of separation between groups. Sham animals clustered on the negative side, whereas the CKD group shifted toward positive values, capturing the dominant multivariate signature associated with CKD-related alterations. PC2 accounted for an additional 28.0% of the variance and reflected intragroup variability without compromising group separation, with both components explaining 76.7% of the total variance. Consistently, correlation analysis indicated that oxidative stress and inflammation were associated with renal dysfunction, as increased lipid and protein oxidation correlated with higher urea and creatinine levels. Furthermore, *NF-*κ*B* expression and *Nrf2* downregulation were associated with a pro-inflammatory and antioxidant-deficient profile. The biplot further demonstrated a clear separation between the Sham and CKD groups, with the CKD group associated with oxidative stress and inflammatory markers and the Sham group associated with higher cardiac *Nrf2* expression. Notably, *Bach1* expression did not correlate with the CKD group, indicating that this preclinical model is not associated with changes in its gene expression.

In conclusion, this study demonstrates that CKD promotes systemic oxidative stress and renal fibrosis, accompanied by reduced cardiac Nrf2 expression. Although renal Bach1 expression remained unchanged, future studies should explore its post-transcriptional regulation and the effects of Bach1 inhibition on Nrf2 signaling. Overall, these findings highlight the therapeutic potential of targeting redox-related pathways, particularly through Nrf2 activation, to mitigate CKD-associated tissue damage.

## Data Availability

The datasets supporting this study’s findings are available from the corresponding author upon reasonable request. To obtain additional details or access to the data, please contact milenabarcza@id.uff.br.
