# Supplementary material for: Renal expression of Bach1 and oxidative stress in chronic kidney disease: evidence from a preclinical model
Source: J Bras Nefrol. 2026 Jun 12;48(3):e20250333. doi: 10.1590/2175-8239-JBN-2025-0333en (PMC13282137; doi:10.1590/2175-8239-JBN-2025-0333en)
Supplement: Translation

# Translation: Expressão renal de Bach1 e estresse oxidativo na doença renal crônica: evidências de um modelo pré-clínico

**DOI:** 10.1590/2175-8239-JBN-2025-0333pt

**Authors:** Beatriz Oliveira Da Cruz, Patricia Pereira Almeida, Nathalia Silva-Costa, Michele Lima Brito, D’Angelo Carlo Magliano, Milena Barcza Stockler-Pinto

**Affiliations:** 1 Universidade Federal Fluminense, Programa de Pós-Graduação em Ciências Cardiovasculares, Niterói, RJ, Brasil.; 2 Universidade Federal Fluminense, Programa de Pós-Graduação em Patologia, Niterói, RJ, Brasil.

**Keywords:** Nefropatias, Estresse Oxidativo, Elementos de Resposta Antioxidante, Nefrectomia.

## Abstract

**Introdução:**

O fator de transcrição BTB e homologia CNC 1 (Bach1) reprime o fator nuclear eritroide 2 relacionado ao fator 2 (Nrf2), que controla a expressão de genes antioxidantes, e seu papel na doença renal crônica (DRC) ainda não está totalmente elucidado.

**Métodos:**

A DRC foi induzida por nefrectomia 5/6 em ratos. Foram mensurados marcadores de fibrose renal e estresse oxidativo. A expressão gênica de Nrf2, Bach1 e fator nuclear kappa B (NF-κB) foi avaliada.

**Resultados:**

A DRC aumentou os marcadores de estresse oxidativo no plasma, no rim e no coração, além de promover fibrose renal. Além disso, a doença reduziu a expressão cardíaca de Nrf2. Entretanto, Bach1, Nrf2 e NF-κB permaneceram inalterados no rim.

**Conclusão:**

A DRC não modulou os níveis de mRNA de Bach1 no rim de ratos submetidos à nefrectomia 5/6.

## INTRODUÇÃO

A geração de espécies reativas de oxigênio (ERO) na doença renal crônica (DRC) contribui para a fisiopatologia da doença por meio de lesão direta das células renais, levando à disfunção, inflamação e fibrose, bem como pela ativação de vias de sinalização e fatores de transcrição que amplificam esses processos^1^. O fator de transcrição BTB e homologia CNC 1 (Bach1) é amplamente expresso e atua como um regulador-chave da resposta ao estresse oxidativo^2^. O Bach1 inibe o fator nuclear eritroide 2 relacionado ao fator 2 (Nrf2) ao competir pelo elemento de resposta antioxidante, suprimindo, assim, a expressão de genes antioxidantes^2^,^3^ e contribuindo para o aumento das ERO, da apoptose e da senescência celular^4^. Enquanto o Nrf2 medeia respostas citoprotetoras, o Bach1 atua como seu principal regulador negativo. Além disso, o equilíbrio entre estresse oxidativo e inflamação é regulado pela interação entre as vias do fator nuclear kappa B (NF-κB) e do Nrf2, uma vez que a ativação de Nrf2 pode atenuar a inflamação mediada por NF-κB, enquanto a ativação de NF-κB pode suprimir a resposta antioxidante^5^. O aumento de Bach1 associado ao envelhecimento prejudica a homeostase redox e agrava o estresse oxidativo. Na DRC, reduções na expressão de Nrf2 e inflamação crônica são frequentemente observadas. Assim, a inibição de Bach1 pode potencializar a atividade de Nrf2 e representar uma estratégia terapêutica promissora^2^,^3^,^4^,^5^,^6^.

Dessa forma, o presente estudo avaliou o estresse oxidativo, os marcadores inflamatórios e as alterações estruturais renais em um modelo pré-clínico de DRC.

## MÉTODOS

Todos os procedimentos foram conduzidos de acordo com as diretrizes ARRIVE e em conformidade com os critérios estabelecidos pelo Conselho Nacional de Controle de Experimentação Animal (CONCEA), sendo aprovados pela Comissão de Ética em Pesquisa Animal da Universidade Federal Fluminense (protocolo nº 956/2017).

### Animais

Foram utilizados quatorze ratos *Wistar* machos, mantidos sob condições controladas de temperatura (22 ± 1 °C) e umidade (60 ± 10%), com ciclo claro-escuro de 12:12 h. Os animais tiveram livre acesso à ração comercial e à água, sendo pesados semanalmente. A DRC foi induzida por nefrectomia 5/6, conforme previamente descrito^7^. Eles foram distribuídos aleatoriamente em dois grupos: controle (Sham), submetido apenas à manipulação cirúrgica dos pedículos renais, sem nefrectomia (n = 7), e DRC, submetido à nefrectomia 5/6 (n = 7).

### Eutanásia

Os animais foram anestesiados com cetamina (40 mg/kg) e xilazina (8 mg/kg) (Laboratório Virbac S.A., São Paulo, Brasil) e eutanasiados por punção cardíaca até exsanguinação total. Os tecidos renais e cardíacos foram cuidadosamente dissecados e armazenados a -80 °C para análises posteriores.

### Análises Bioquímicas

As amostras de sangue foram obtidas por punção cardíaca. O plasma e o soro foram separados por centrifugação (15 min, 4500 × g, 4 °C). As concentrações séricas de creatinina e ureia foram mensuradas utilizando kits Bioclin^®^ por meio do analisador bioquímico Bioclin BS-120.

### Peroxidação Lipídica e Carbonilação de Proteínas

A peroxidação lipídica nos tecidos renal e cardíaco foi avaliada pelo ensaio de substâncias reativas ao ácido tiobarbitúrico (TBARS), e a oxidação proteica plasmática foi determinada pela quantificação de grupos carbonila usando 2,4-dinitrofenilhidrazina (DNPH). Ambas as análises seguiram a metodologia descrita por Costa et al.^8^. Todas as amostras foram preparadas em tampão RIPA contendo coquetel inibidor de protease (*Sigma-Aldrich*, Missouri, EUA).

### Morfologia Renal

Os cortes renais foram corados com tricrômico de Masson para avaliação da fibrose túbulo-intersticial. Dez campos aleatórios da área túbulo-intersticial de cinco animais foram selecionados e analisados utilizando o software *Image-Pro Plus* (*Media Cybernetics*, Silver Spring, MD, EUA), por meio da ferramenta de seleção por limiar de densidade.

### Expressão Gênica

As expressões de mRNA de *Bach1*, *NF-*κ*B* e *Nrf2* foram avaliadas por reação em cadeia polimerase (do inglês, polymerase chain reaction – PCR) quantitativo em tempo real, conforme Leal et al.^9^. Foram utilizados ensaios *TaqMan Gene Expression* (*Thermo Fisher Scientific*) para *Bach1* (Rn01477344_m1), *NF-*κ*B* (Rn01399572_m1), *Nrf2* (Rn00477784_m1) e o gene controle *Gapdh* (Rn01775763_g1). As reações foram realizadas no sistema *ABI Prism 7500 Sequence Detection System* (*Applied Biosystems*) sob condições padrão. A expressão de *Bach1* foi avaliada apenas no tecido renal, e as de *NF-*κ*B* e *Nrf2*, no rim e no coração.

### Análises Estatísticas

Os resultados são apresentados como média ± desvio-padrão, e o teste ROUT foi utilizado para identificação de *outliers*. Avaliou-se a normalidade dos dados por meio do teste de *Shapiro–Wilk*. Comparações entre grupos foram realizadas pelo teste t não pareado, e a ANOVA de duas vias foi aplicada para análises ao longo do tempo. Também adotou-se uma abordagem multivariada. A variação no tamanho da amostra para expressão gênica refletiu a disponibilidade limitada de material biológico. Uma matriz de correlação foi gerada, e a análise de componentes principais (PCA) foi conduzida sobre variáveis padronizadas (transformação z *score*), a fim de equalizar escalas de medida e garantir contribuição igualitária das variáveis. A retenção de componentes seguiu o critério de *Kaiser–Guttman*, considerando-se significativos valores próprios (*eigenvalues*) ≥ 1,0, e a variância explicada por cada componente retido foi reportada. A PCA constituiu um método exploratório e não supervisionado de redução de dimensionalidade para identificar padrões dominantes e relações entre variáveis correlacionadas, facilitando a interpretação e visualização dos dados, em vez de testes inferenciais. Todas as análises foram realizadas no *GraphPad Prism* (versão 10.2.3; *GraphPad Software*, Boston, MA, EUA), considerando-se significância estatística em p < 0,05.

## RESULTADOS

Após 4 semanas, a DRC foi estabelecida. O grupo DRC apresentou massa corporal significativamente menor do que o grupo controle (Figura 1A). Como esperado, o grupo DRC apresentou aumento significativo na creatinina (1,06 ± 0,14 vs. 0,63 ± 0,05 mg/dL; p = 0,0006) e nos níveis séricos de ureia em comparação ao grupo controle (60,29 ± 5,70 vs. 30,29 ± 2,98 mg/dL; p < 0,0001) (Figuras 1B e 1C). Além disso, a carbonilação proteica plasmática esteve significativamente elevada no grupo DRC em comparação ao grupo controle (689,90 ± 42,06 vs. 584,80 ± 27,73 nmol/mg proteína; p = 0,0003) (Figura 1D). Quanto à peroxidação lipídica, os ratos DRC exibiram níveis marcadamente maiores no rim (0,27 ± 0,12 vs. 0,14 ± 0,02 μmol/mg tecido; p = 0,0451) e no coração (2,46 ± 0,69 vs. 0,77 ± 0,40 μmol/mg tecido; p = 0,0055) em comparação ao grupo controle (Figuras 1E e 1F). A análise histológica das fotomicrografias renais revelou aumento pronunciado da fibrose túbulo-intersticial no grupo DRC em relação aos controles (Figura 1G).

**Figura 1:** Evolução da massa corporal ao longo de 12 semanas nos grupos controle e DRC (A). Creatinina sérica (B), ureia sérica (C) e carbonilação proteica (D). Níveis de Peroxidação lipídica no rim (E) e no coração (F) de animais controle e DRC. Fotomicrografias representativas do tecido renal de animais controle e DRC (G). Análise histológica utilizando coloração tricrômica de Masson (×400) para avaliar fibrose túbulo-intersticial. Setas (↑) indicam deposição de colágeno, marcadamente aumentada no grupo DRC em comparação ao grupo controle. (A), (F) e (G): controle: n = 5; DRC: n = 5; (B) e (C): controle: n = 7; DRC: n = 7; (D): controle: n = 6; DRC: n = 7; (E): controle: n = 4; DRC: n = 4.

Abreviaturas – DRC: doença renal crônica.

Notas – Dados apresentados como média ± DP. ANOVA de duas vias seguida do teste *post hoc* de *Bonferroni* foi utilizada para a evolução da massa corporal; teste t não pareado (dados paramétricos) foi aplicado para os demais parâmetros.

Não houve diferença significativa na expressão de mRNA de *Bach1* no rim entre os grupos (Figura 2A). A expressão de mRNA de *NF-*κ*B* permaneceu inalterada tanto no coração (p = 0,9982) quanto no rim (p = 0,7117) (Figuras 2B e 2C). Entretanto, os animais do grupo DRC apresentaram menor expressão de mRNA de *Nrf2* no coração (0,71 ± 0,07 vs. 1,03 ± 0,24; p = 0,0241) em comparação ao grupo controle (Figura 2D); não se observou diferença no rim (1,46 ± 1,53 vs. 0,92 ± 0,67; p = 0,3432) (Figura 2E).

**Figura 2:** Expressão de mRNA de *Bach1* no rim (A), *NF*-κ*B* no coração (B), *NF*-κ*B* no rim (C), *Nrf2* no coração (D) e *Nrf2* no rim (E) dos grupos experimentais.

Abreviaturas – Bach1: fator de transcrição BTB e homologia CNC 1; NF-κB: fator nuclear kappa B; e Nrf2: fator nuclear eritroide 2 relacionado ao fator 2. DRC: doença renal crônica.

Notas – Significância estatística: p < 0,05. Teste t de *Student* não pareado; *outliers* identificados pelo método ROUT foram excluídos da análise estatística. (A): controle: n = 5; DRC: n = 4; (B): controle: n = 7; DRC: n = 6; (C): controle: n = 6; DRC: n = 6; (D): controle: n = 4; DRC: n = 5; (E): controle: n = 5; DRC: n = 5.

A análise multivariada foi realizada considerando parâmetros relacionados à inflamação, estresse oxidativo e função renal. A matriz de correlação é apresentada como mapa de calor na Figura 3A. Por sua vez, a PCA resultou em dois componentes principais, explicando 48,7% (PC1) e 28,0% (PC2) da variância, totalizando 76,7% da variância acumulada (Figura 3B). O PC1 foi fortemente correlacionado com a expressão renal de *Bach1*, *NF-*κ*B* e *Nrf2*, indicando uma resposta integrada entre essas variáveis. O PC2 associou-sepredominantemente às concentrações de peroxidação lipídica renal e às expressões cardíacas de *Bach1*, *NF-*κ*B* e *Nrf2* (Figuras 3C e 3D).

**Figura 3:** Análise de componentes principais. Mapa de calor da correlação de *Pearson* (A). Variância acumulada dos CPs (B). *Biplot* das cargas (C). Gráfico dos escores dos CPs (D). Controle: n = 5; DRC: n = 5.

Abreviaturas – DRC: doença renal crônica; CP: componente principal.

## DISCUSSÃO

Os resultados deste estudo demonstram que a DRC em ratos está associada ao aumento do estresse oxidativo, evidenciado pela elevação da peroxidação lipídica nos tecidos renal e cardíaco e pelo aumento dos níveis plasmáticos de carbonilação proteica. Além disso, os animais do grupo DRC exibiram redução da expressão de mRNA de Nrf2 no tecido cardíaco, sugerindo uma resposta antioxidante sistêmica comprometida. Notavelmente, a expressão de mRNA de Bach1 no rim não apresentou diferença significativa entre os grupos, apesar de seu papel estabelecido como regulador negativo de Nrf2.

Considerando que a ativação da via Nrf2 constitui um alvo terapêutico na DRC, sua regulação negativa tem sido associada à progressão de distúrbios cardiometabólicos^10^,^11^. A regulação de Nrf2 envolve mecanismos moleculares complexos, influenciados por múltiplos processos fisiopatológicos^12^. No presente estudo, os níveis de transcrição de Nrf2 diferiram entre tecidos, com redução no coração, mas não no rim, indicando uma regulação tecidual específica, que pode estar associada ao aumento da peroxidação lipídica e da carbonilação proteica.

Apesar da presença da DRC e do estresse oxidativo e da inflamação associados em nosso modelo experimental, não foram observadas diferenças significativas na expressão gênica renal de Bach1 entre os grupos. Esse achado foi inesperado, dado que Bach1 é um repressor transcricional sensível ao redox, conhecido por responder ao estresse oxidativo regulando elementos de resposta antioxidante, frequentemente atuando em oposição a Nrf2^4^,^13^. Estudos prévios demonstraram que, sob condições de estresse oxidativo agudo, como lesão renal aguda associada à rabdomiólise, a expressão e a localização subcelular de Bach1 podem mudar dinamicamente, com exportação nuclear representando uma resposta precoce a níveis elevados de heme^14^. Em contraste, sob condições crônicas, como a DRC, a regulação de Bach1 pode diferir e ser influenciada por mecanismos adaptativos ou compensatórios que ajudam a manter níveis de expressão estáveis, apesar do estresse oxidativo sustentado^3^,^15^. Assim, a ausência de alterações transcricionais em Bach1 não invalida sua relevância funcional, uma vez que sua atividade pode ser modulada por mecanismos pós-transcricionais e pós-translacionais, incluindo alterações na estabilidade proteica ou na localização subcelular^16^. Embora a interação Bach1–Nrf2 possa ser regulada dinamicamente ao longo do tempo, este estudo foi limitado a um único ponto temporal experimental, e qualquer interpretação temporal deve, portanto, ser feita com cautela. Ademais, no modelo de nefrectomia 5/6, observa-se aumento da fibrose túbulo-intersticial, juntamente com hiperfiltração adaptativa e hipertrofia glomerular dos néfrons residuais para sustentar a capacidade excretora^17^. Essas mudanças compensatórias aumentam a pressão intraglomerular e o estresse mecânico, promovendo lesão da barreira de filtração, estresse oxidativo e ativação de vias pró-inflamatórias^18^. É importante salientar que a atividade inflamatória pode ocorrer sem alterações na expressão gênica de NF-κB, visto que a ativação de NF-κB é amplamente regulada ao nível pós-translacional^19^. Coletivamente, esses mecanismos podem ter influenciado a expressão renal de Bach1 e comprometido respostas antioxidantes, incluindo Nrf2.

Por fim, a análise por PCA mostrou que o PC1 explicou 48,7% da variância total e representou o principal eixo de separação entre os grupos. Os animais Sham agruparam-se no lado negativo, enquanto o grupo DRC se deslocou para valores positivos, capturando a assinatura multivariada dominante associada às alterações relacionadas à DRC. O PC2 respondeu por um adicional de 28,0% da variância e refletiu variabilidade intragrupo sem comprometer a separação entre grupos, com ambos os componentes explicando 76,7% da variância total. Consistentemente, a análise de correlação indicou que o estresse oxidativo e a inflamação associaram-se à disfunção renal, à medida que o aumento da oxidação lipídica e proteica se correlacionou com concentrações mais elevadas de ureia e creatinina, enquanto a expressão de *NF-*κ*B* e a regulação negativa de *Nrf2* se relacionaram a um perfil pró-inflamatório e com deficiência antioxidante. O *biplot* demonstrou claramente a separação entre os grupos Sham e DRC, com o grupo DRC associado a marcadores de estresse oxidativo e inflamatórios e o grupo Sham associado à maior expressão cardíaca de *Nrf2*. Notavelmente, a expressão de *Bach1* não se correlacionou com o grupo DRC, indicando que este modelo pré-clínico não está associado a alterações em sua expressão gênica.

Em conclusão, este estudo demonstra que a DRC promove estresse oxidativo sistêmico e fibrose renal, acompanhados de redução da expressão cardíaca de *Nrf2*. Embora a expressão renal de *Bach1* tenha permanecido inalterada, estudos futuros devem explorar sua regulação pós-transcricional e os efeitos da inibição de Bach1 na sinalização de Nrf2. Em conjunto, esses achados enfatizam o potencial terapêutico de direcionar vias relacionadas ao redox, particularmente por meio da ativação de Nrf2, para mitigar danos teciduais associados à DRC.

## Data Availability

Os conjuntos de dados que sustentam as conclusões deste estudo estão disponíveis mediante solicitação razoável ao autor correspondente. Para obter detalhes adicionais ou acesso aos dados, contate milenabarcza@id.uff.br.
